# Supplementary material for: Transcriptional and morphological profiling of parvalbumin interneuron subpopulations in the mouse hippocampus
Source: Nat Commun. 2021 Jan 4;12:108. doi: 10.1038/s41467-020-20328-4 (PMC7782706; doi:10.1038/s41467-020-20328-4)
Supplement: Supplementary file 1 — Supplementary Information [file 41467_2020_20328_MOESM1_ESM.pdf]

Supplementary Information

Transcriptional and morphological profiling of parvalbumin interneuron subpopulations in the mouse hippocampus

Lin Que[1], David Lukacsovich[1], Wenshu Luo[1] and Csaba Földy[1]

[1]Laboratory of Neural Connectivity, Brain Research Institute,
Faculties of Medicine and Science, University of Zürich, Switzerland

Supplementary Figures:

Fig. S1. Morphological reconstruction of different PV types. (Related to Fig. 1.)

Fig. S2. Single-cell RNAseq profiling of PV-INs. (Related to Fig. 1.)

Fig. S3. Transcriptomic clustering at low sample numbers. (Related to Fig. 1 and 4.)

Fig. S4. Transcriptomic characterization of PV-INs. (Related to Fig. 2.)

Fig. S5. Cross-comparison of electrophysiological parameters measured in PV-INs. (Related to Fig. 3.)

Fig. S6. Support vector machine classification and gene selection in morphological PV types. (Related to Fig. 4.)

Fig. S7. Expression of morphology-associated genes in the CA1-IN data set[1]. (Related to Fig. 4.)

Fig. S8. Electrophysiological analysis of vBC type PV-INs during circuit maturation. (Related to Fig. 6.)

Fig. S9. Transcriptomic analysis of vBC type PV-INs during circuit maturation. (Related to Figs. 6-7.)

Fig. S10. Age-dependent gene expression changes in PV-INs. (Related to Fig. 7.)

Fig. S11. Detection of hemoglobin mRNA expression in publicly available single-cell RNA datasets. (Related to Fig. 7.)

Fig. S12. Hemoglobin subunit expression at single-nucleotide level. (Related to Fig. 7.)

Supplementary Data Sheet:

Que_et_al.xlsx This file contains all numerical information referenced in the manuscript. (Related to Figs. 1-7 and S1-11.)

Figure S1

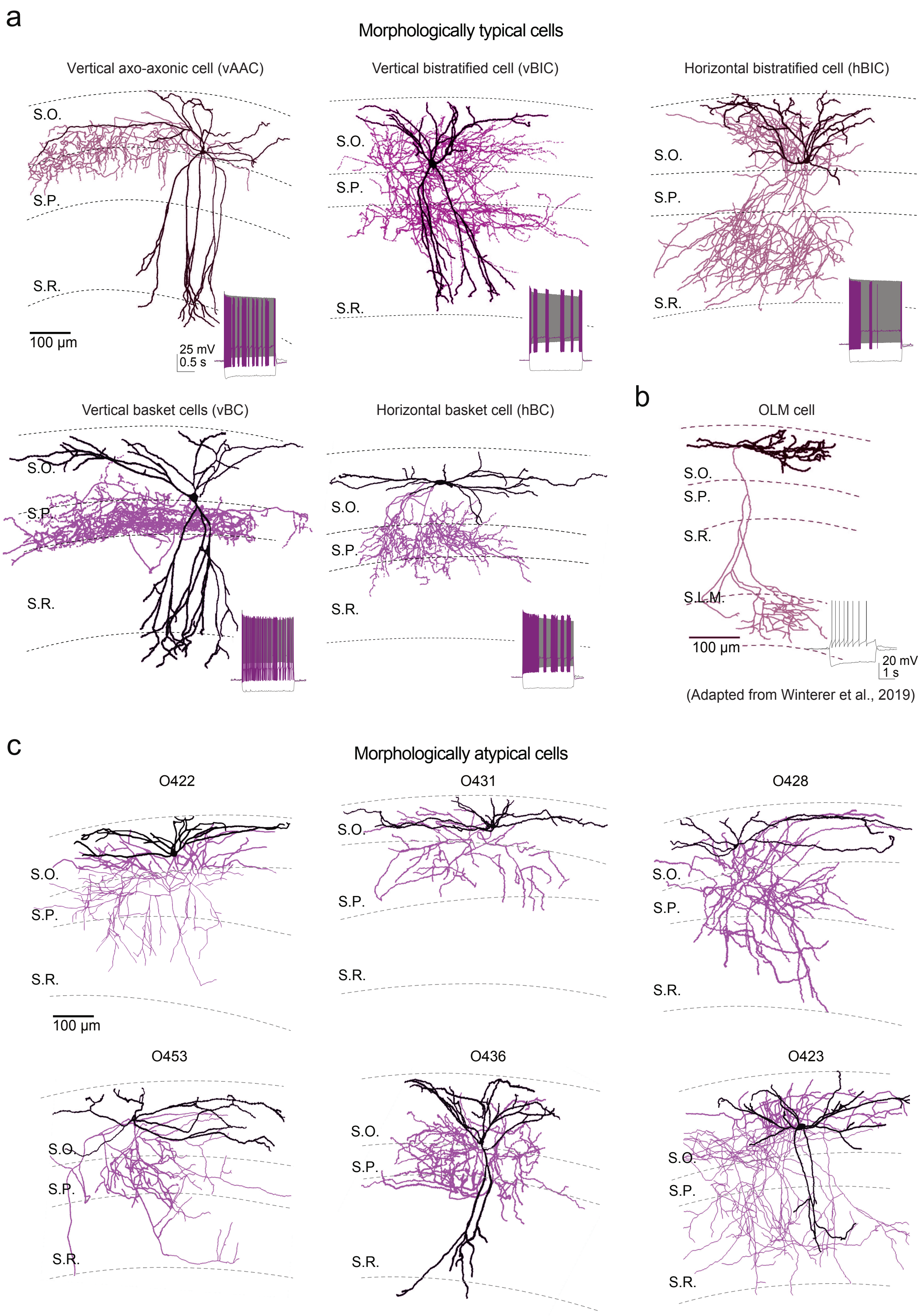
a
Morphologically typical cells
Vertical axo-axonic cell (vAAC)
Vertical bistratified cell (vBIC)
Horizontal bistratified cell (hBIC)
S.O.
S.P.
S.R.
100 µm
25 mV
0.5 s
Vertical basket cells (vBC)
Horizontal basket cell (hBC)
b
OLM cell
S.L.M.
20 mV
1 s
(Adapted from Winterer et al., 2019)
c
Morphologically atypical cells
O422
O431
O428
O453
O436
O423

Fig. S1. Morphological reconstruction of different PV types. a. Example cells are shown for each morphological PV type together with their response to hyperpolarizing and depolarizing current pulses (traces in the bottom right). Scales shown for axo-axonic cell apply to all. **b.** Example OLM cell. **c.** Example cells that could not be unambiguously classified as either of vAAC, vBIC, hBIC, vBC or hBC type. O422 and O431: insufficient axon recovery could not support classification as either hBIC or vBIC type. O428, O453 and O436: these cells display apparent trilaminar axonal morphology, which could not be adjusted for with an angular adjustment in 3D reconstructions (see Methods). O423: This one cell displayed both vertical and horizontal dendrites, causing ambiguity in its classification.

Figure S2

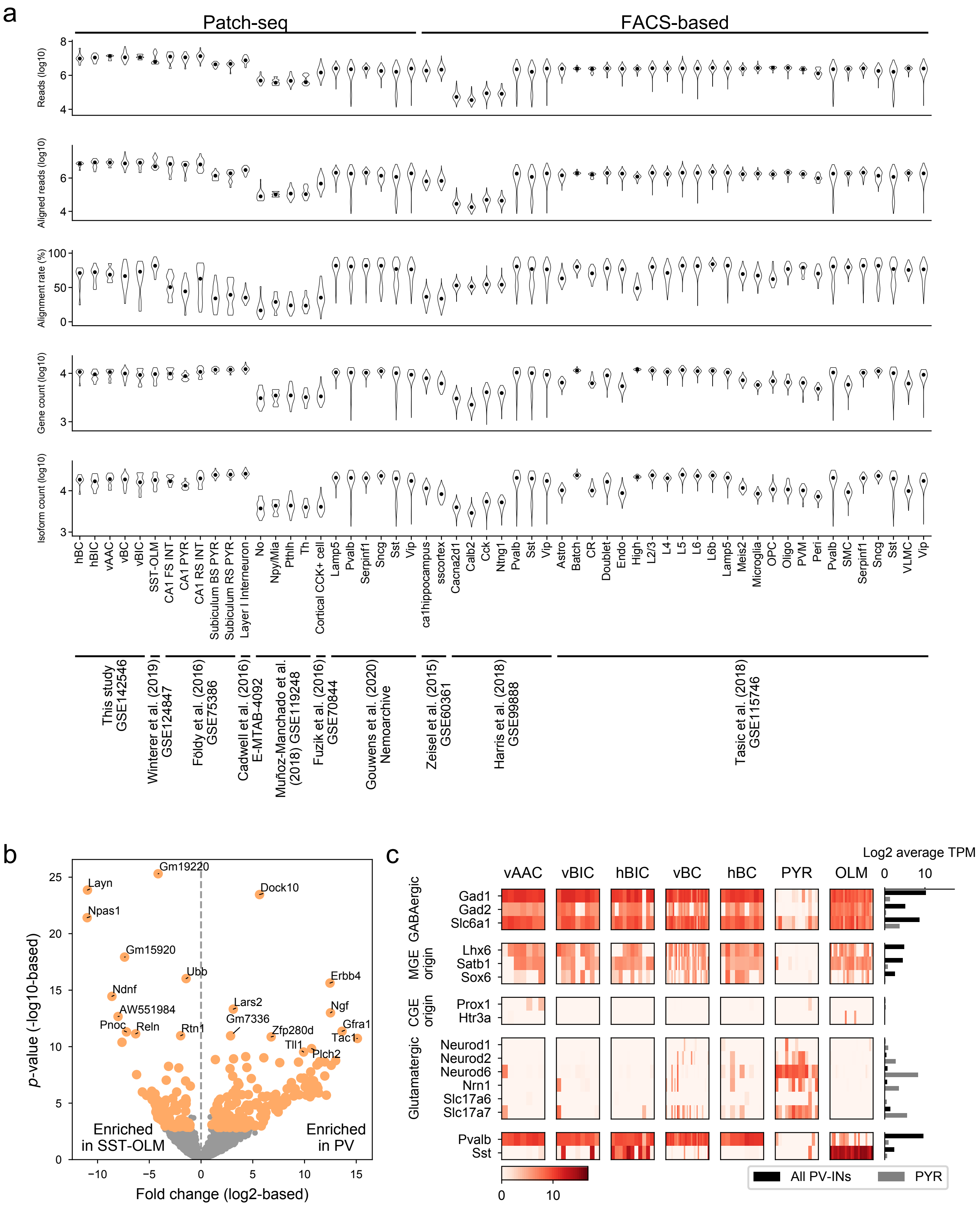
a
Patch-seq
FACS-based
Reads (log10)
Aligned reads (log10)
Alignment rate (%)
Gene count (log10)
Isoform count (log10)
hBC
hBIC
vAAC
vBC
vBIC
SST-OLM
CA1 FS INT
CA1 PYR
CA1 RS INT
Subiculum BS PYR
Subiculum RS PYR
Layer I Interneuron
No
Npy/Mia
Pthlh
Th
Cortical CCK+ cell
Lamp5
Pvalb
Serpinf1
Sncg
Sst
Vip
ca1hippocampus
sscortex
Cacna2d1
Calb2
Cck
Ntng1
Pvalb
Sst
Vip
Astro
Batch
CR
Doublet
Endo
High
L2/3
L4
L5
L6
L6b
Lamp5
Meis2
Microglia
OPC
Oligo
PVM
Peri
Pvalb
SMC
Serpinf1
Sncg
Sst
VLMC
Vip
This study GSE142546
Winterer et al. (2019) GSE124847
Földy et al. (2016) GSE75386
Cadwell et al. (2016) E-MTAB-4092
Muñoz-Manchado et al. (2018) GSE119248
Fuzik et al. (2016) GSE70844
Gouwens et al. (2020) Nemoarchive
Zeisel et al. (2015) GSE60361
Harris et al. (2018) GSE99888
Tasic et al. (2018) GSE115746
b
p-value (-log10-based)
Fold change (log2-based)
Layn
Gm19220
Dock10
Npas1
Gm15920
Ubb
Erbb4
Ndnf
AW551984
Lars2
Ngf
Gm7336
Gfra1
Pnoc
Reln
Rtn1
Zfp280d
Tac1
Tll1
Plch2
Enriched in SST-OLM
Enriched in PV
c
vAAC
vBIC
hBIC
vBC
hBC
PYR
OLM
Log2 average TPM
GABAergic
Gad1
Gad2
Slc6a1
MGE origin
Lhx6
Satb1
Sox6
CGE origin
Prox1
Htr3a
Glutamatergic
Neurod1
Neurod2
Neurod6
Nrn1
Slc17a6
Slc17a7
Pvalb
Sst
All PV-INs
PYR

Fig. S2. Single-cell RNA-seq profiling of PV-INs. a. Violin plots show quality control parameters (number of reads, aligned reads, alignment rate, gene count, and isoform count) after single-cell sequencing for 5 morphological PV and for other publicly available data sets, for comparison. **b.** We first made comparisons between this current PV set to a previously generated SST-OLM interneuron data set, which was adopted from Winterer et al.[2] and generated with identical methods as the PV set of this study. We hypothesized that transcriptomic differences between PV and SST cells would define an 'upper limit' of molecular distinctions that may be present between the different morphological PV types. Therefore, before examining differential gene expression between morphological PV types in detail, we analyzed differences between PV and SST cells. Volcano plot shows differential gene expression between PV, where all morphological types were pooled together, and SST-OLM cells. Each circle represents a single gene. Orange color denotes genes that are differentially expressed between the two cell types with a 2-fold difference ($|\log2|>1$) and an $FDR<0.05$. Statistics were calculated using edgeR. The number of differentially enriched genes are n=93 and 168 in SST-OLM and PV-INs, respectively. **c.** Heat map shows expression of GABAergic, MGE, CGE, and glutamatergic marker genes in single PV-INs (grouped by their morphological type), pyramidal (PYR, from Földy et al.[3]) and SST-OLM cells. On the right, bar plot shows the expression level of each gene, averaged across all PV-INs (black bars), independent of their morphological type. Gray bars show average values for glutamatergic CA1 pyramidal cells (PYR), for comparison.

Figure S3

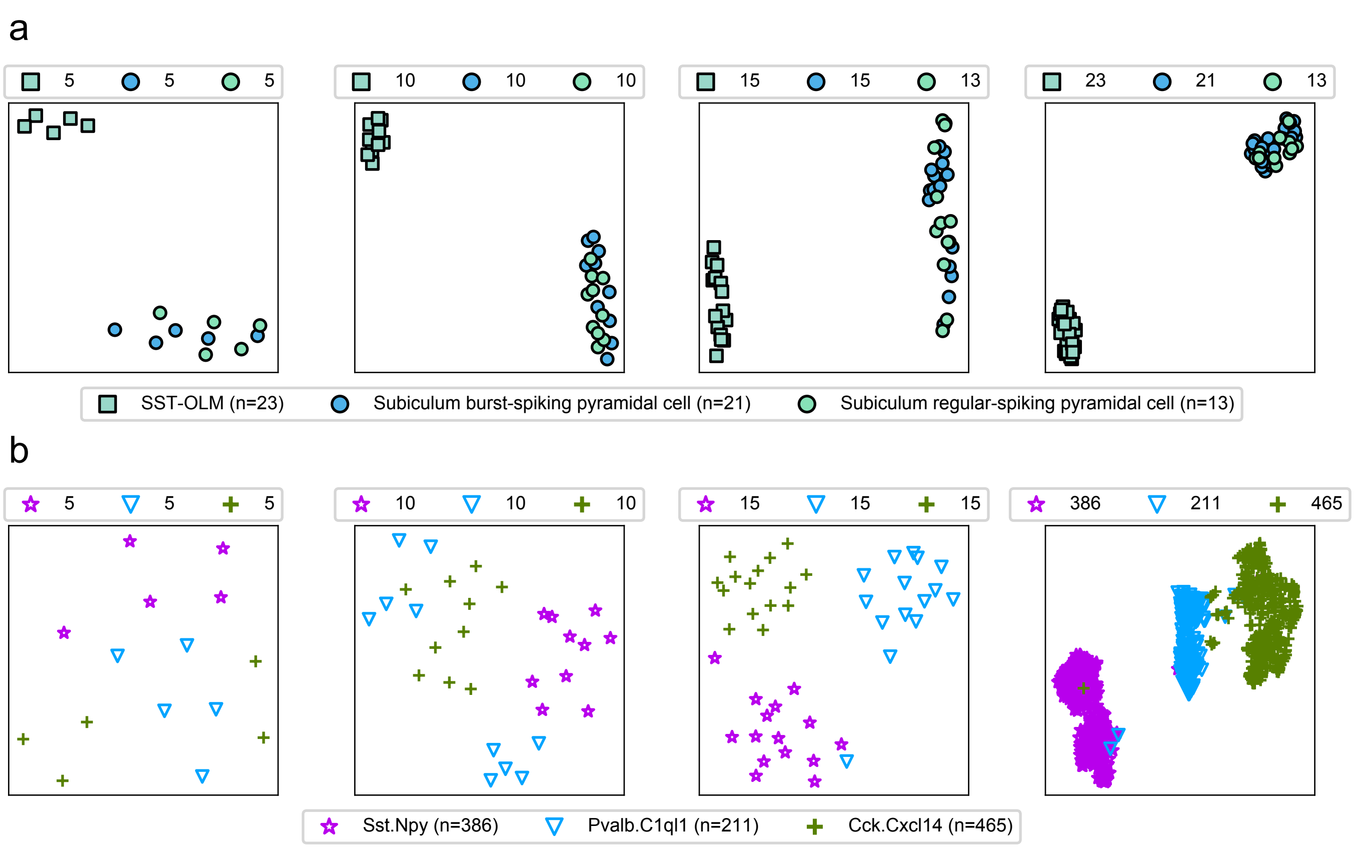

Fig. S3. Transcriptomic clustering at low sample numbers. In this study, our sample numbers for each morphological PV types are in the low 10s (vAAC: 7, vBIC: 9, hBIC: 11, vBC: 31, hBC: 9, and SST-OLM: 23). However, most bioinformatics tools for clustering of single cell RNA-seq data are developed for and tested on sample numbers on the order of 100s or 1,000s. Here, we show that our algorithms (rnaseqTools for feature selection and UMAP for embedding) are functional with a dataset of our size: using the same parameter settings as in Fig. 1d, we validated the efficacy of clustering at low sample numbers. **a.** Transcriptomic clustering of three, electrophysiologically recorded (patch-RNAseq) cell type of which one (SST-OLM; from Winterer et al.[2]) was different from the two others (Subiculum burst-firing and regular-firing pyramidal cells; from Földy et al.[3]), which two were transcriptomically not different from one other[3]. For clustering, we randomly chose 5, 10, 15 (or 13 in the case of subiculum regular firing cells, which was the total sample number) cells from each data set, or used the complete data sets, from left to right, respectively. These showed separate clustering of SST-OLM cells already at the lowest sample numbers (left plot), whereas subiculum pyramidal cells did not separately cluster from one other even when the complete data sets were used (right plot). **b.** Transcriptomic clustering of three, FACS-collected cell types which are all different from one other (*Sst.Npy*, *Pvalb.C1ql1*, and *Cck.Cxcl14*; from Harris et al.[1]). For clustering, we randomly chose 5, 10, 15 cells from each data set, or used the complete data sets, from left to right, respectively. While clustering was less apparent when 5 cells were chosen (left panel), it become more robust already at 10 cells (second panel). Together, these analyses show that the method of choice for this study, robustly clusters cells separately if those are transcriptionally different, whereas similar cells will cluster together even at larger cell numbers.

Figure S4

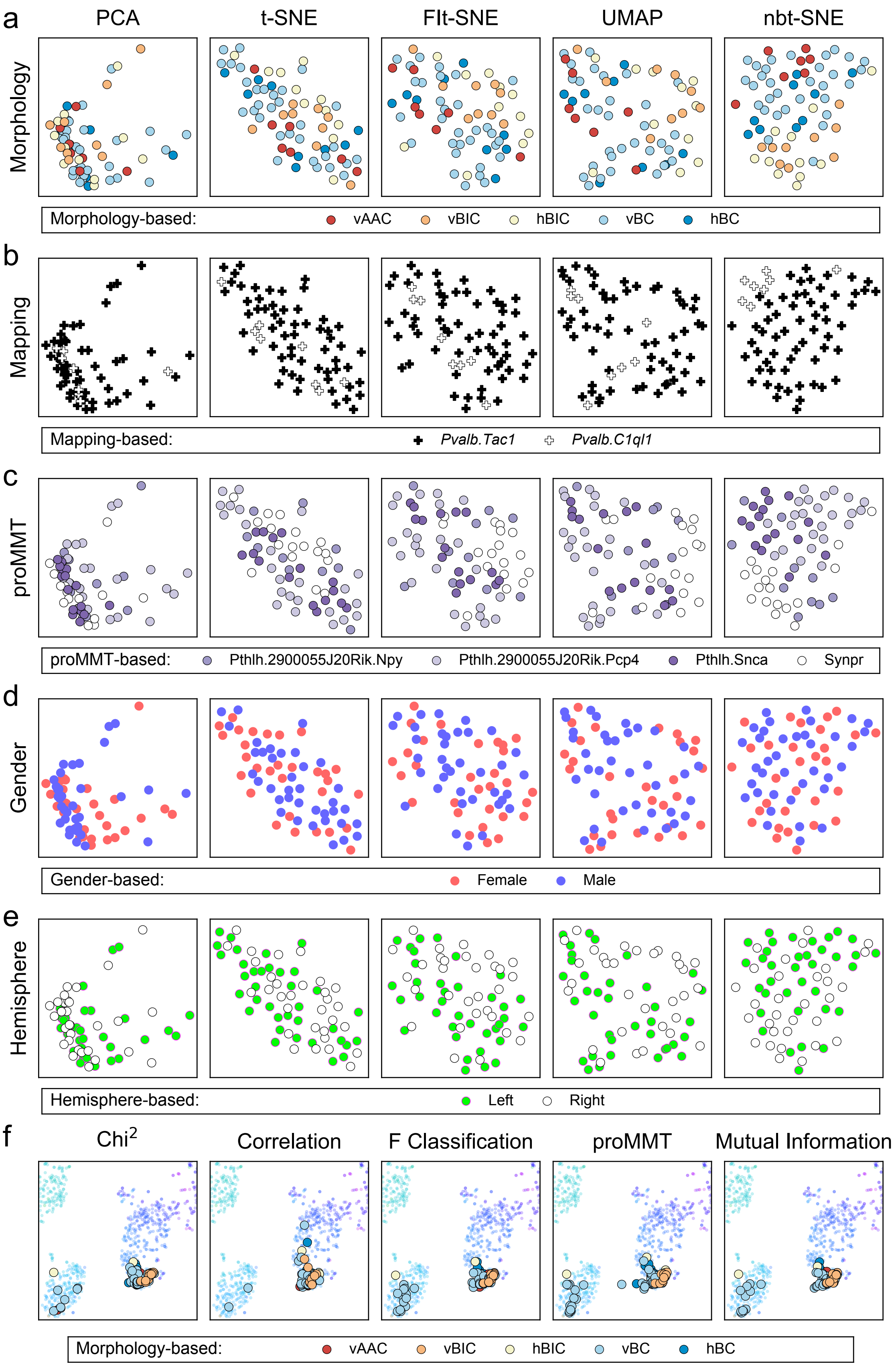
a
PCA
t-SNE
FIt-SNE
UMAP
nbt-SNE
Morphology
Morphology-based: vAAC vBIC hBIC vBC hBC
b
Mapping
Mapping-based: Pvalb.Tac1 Pvalb.C1ql1
c
proMMT
proMMT-based: Pthlh.2900055J20Rik.Npy Pthlh.2900055J20Rik.Pcp4 Pthlh.Snca Synpr
d
Gender
Gender-based: Female Male
e
Hemisphere
Hemisphere-based: Left Right
f
Chi2
Correlation
F Classification
proMMT
Mutual Information
Morphology-based: vAAC vBIC hBIC vBC hBC

Fig. S4. Transcriptomic characterization of PV-INs. a-e. Our transcriptomic characterization of PV-INs using proMMT revealed four clusters, but visualization using nbt-SNE could not clearly separate these clusters in the two-dimensional space (Fig. 2a). Here, using multiple different visualization approaches, we show invariant similarly and lack of separation in transcriptome-based clustering of PV-INs. The five visualization approaches that we used are PCA, t-SNE, Flt-SNE, UMAP, and nbt-SNE, and are labeled on the top of each column. Note that since all plots in a column use the same visualization technique, the location of symbols (representing single cells) are the same. However, in each row, we colored the symbols differently, according to different classification scenarios that we considered. **a.** Cells are labeled according to the 5 morphological PV types. None of the five visualization techniques revealed additional separation in the two-dimensional space than nbt-SNE. **b.** Cells are labeled according to whether they mapped onto *Pvalb.Tac1* or *Pvalb.C1ql1* (see Fig. 2d and e). **c.** Cells are labeled according to their proMMT clusters (the rightmost nbt-SNE plot is shown in Fig. 2a). **d.** Cells are labeled according to the gender of the animal from which they were collected from. **e.** Cells are labeled according to the side of the hemisphere they were collected from (left or right hemisphere). To conclude, these visualizations show a lack of transcriptomic separation based on morphological, gender, or hemispherical properties. **f.** Plots show mapping of our PV-INs onto the PV-INs of the CA1-IN data set[1], using 5 different methods of feature selection; chi-squared, F Classification and Mutual Information were used on the PV-INs from the CA1-INs data[1] set to find the best markers for *Pvalb.Tac1* and *Pvalb.C1ql1* clusters in that data set. proMMT represents clustering based on the 144 genes used in the CA1-IN study which were in the Ensembl reference index used in this study, and Correlation is based on the top 150 genes that best correlated with these 144 genes, but not including those (see also Fig. 2d).

Figure S5

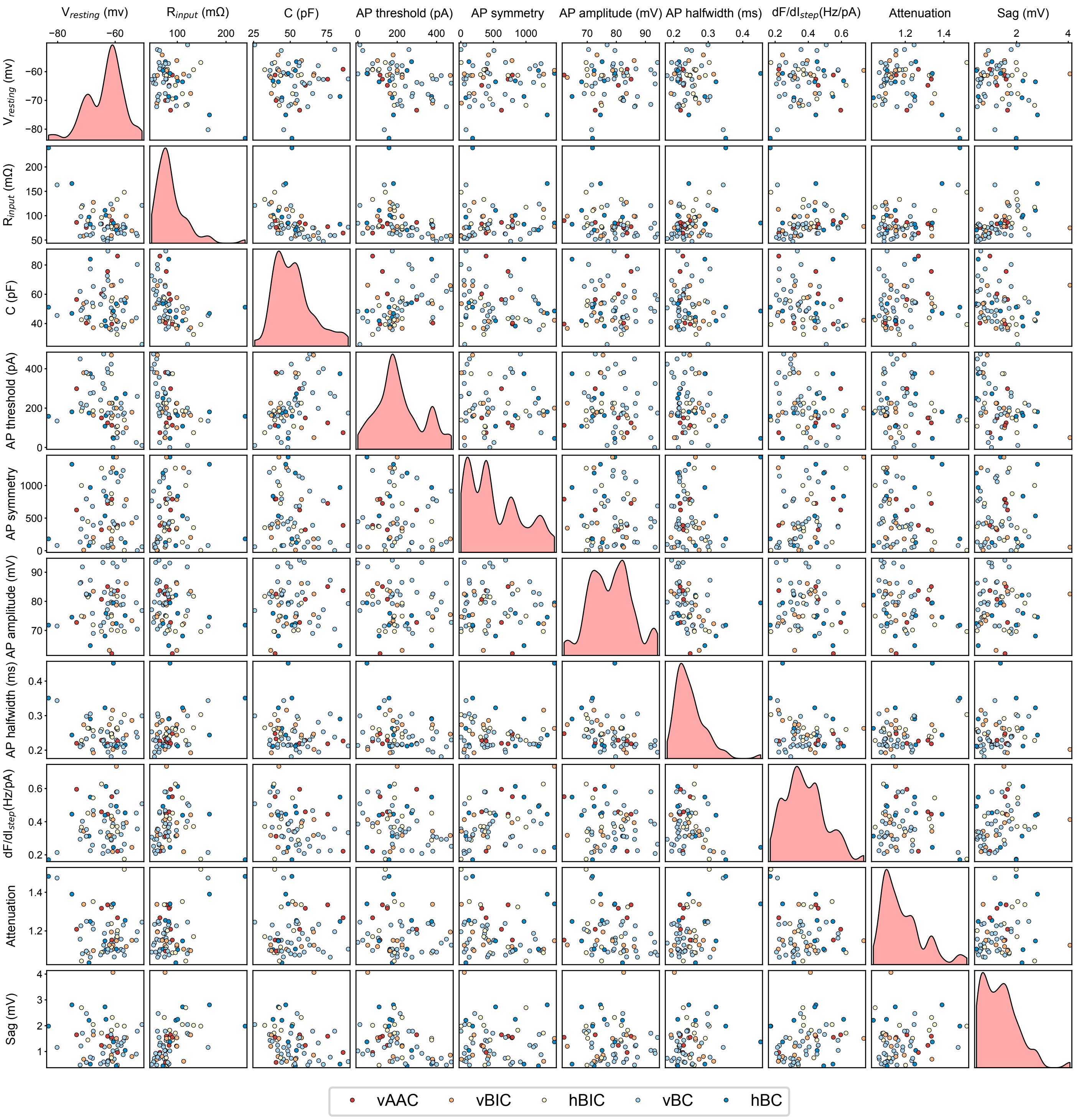

$V_{resting}$ (mv)
R_{input} (mΩ)
C (pF)
AP threshold (pA)
AP symmetry
AP amplitude (mV)
AP halfwidth (ms)
dF/dI$_{step}$(Hz/pA)
Attenuation
Sag (mV)
vAAC
vBIC
hBIC
vBC
hBC

Fig. S5. Cross-comparison of electrophysiological parameters measured in PV-INs. In Fig. 3, we show that unbiased clustering of electrophysiological parameters measured in PV-INs did not reveal biophysical PV types. To support this conclusion, here we considered the possibility that hidden pair-wise correlations between different electrophysiological parameters may define distinct biophysical clusters, not detectable by unbiased clustering. To detect these, we made scatter plots displaying data points of each pair of ten electrophysiological parameters we analyzed. However, none of these plots revealed distinctly separating clusters, indicative of different biophysical types among hippocampal PV-INs. Plots along the diameter display histograms showing the distribution of the corresponding parameter measured in all >P21 PV-INs (n=66) for which all electrophysiological measurements were available, independent of cells' morphological and transcriptomic type.

Figure S6

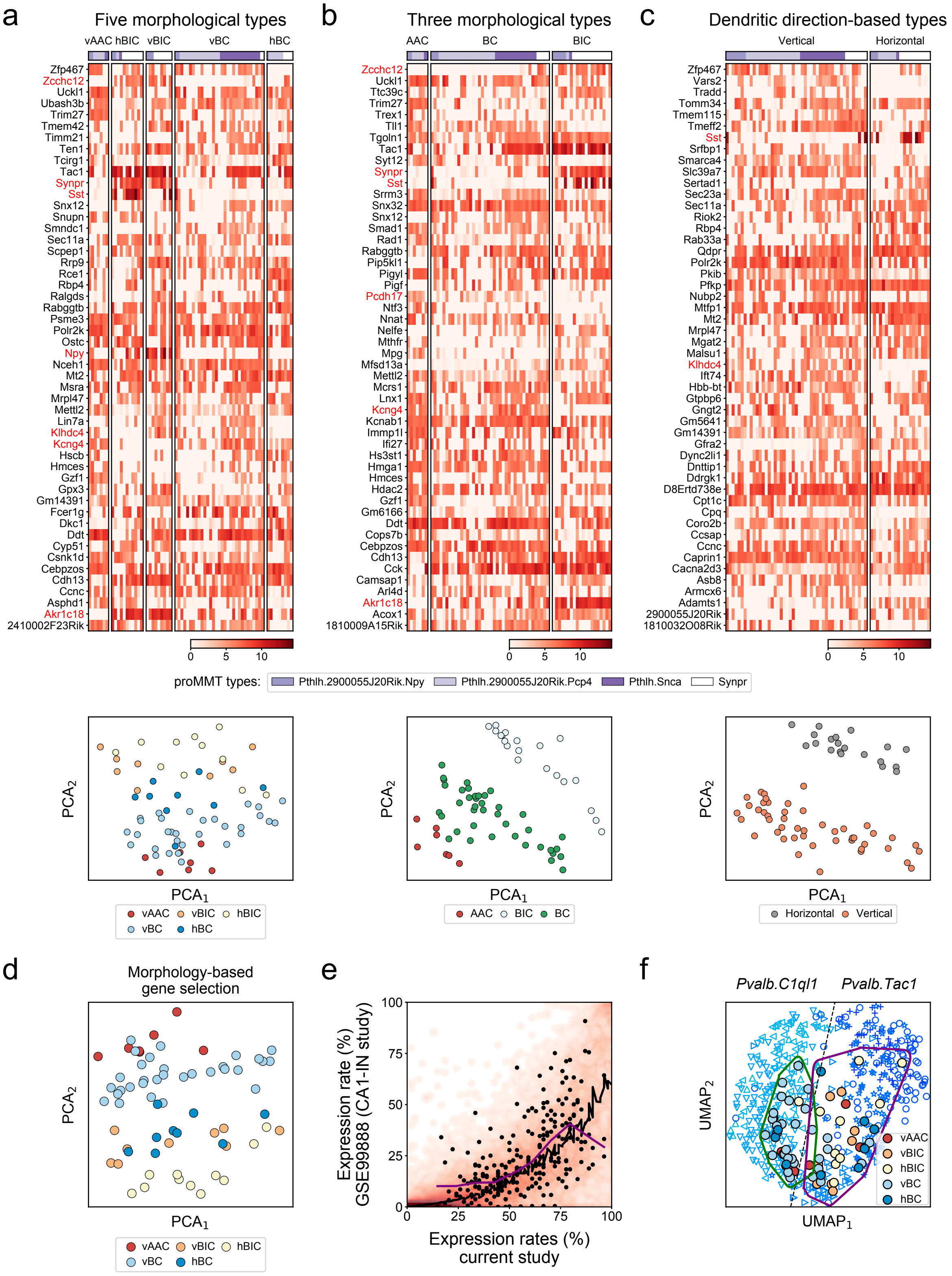

a
Five morphological types
vAAC hBIC vBIC vBC hBC
b
Three morphological types
AAC BC BIC
c
Dendritic direction-based types
Vertical
Horizontal
proMMT types:
Pthlh.2900055J20Rik.Npy
Pthlh.2900055J20Rik.Pcp4
Pthlh.Snca
Synpr
PCA1
PCA2
vAAC vBIC hBIC vBC hBC
AAC BIC BC
Horizontal Vertical
d
Morphology-based gene selection
e
Expression rate (%) GSE99888 (CA1-IN study)
Expression rates (%) current study
f
Pvalb.C1ql1
Pvalb.Tac1
UMAP1
UMAP2

Fig. S6. Support vector machine classification and gene selection in morphological PV types. In Fig. 4, we used statistical comparisons to identify enriched genes among different morphological PV types. Here, we implemented random forest classification to this problem. **a-c.** Heat maps (upper plots) of gene sets identified by linear support vector machine (svm) classification between the 5 morphological PV types (recursive feature elimination was used to select 50 genes; panel a), axo-morphological types (panel b), and dendro-morphological types (panel c). Cells are ordered according to age. Note that individual genes are not necessarily significantly different between any two morphological type. Genes names colored in red are statistically significant (using edgeR, fold difference>2, FDR<0.05) and also appeared in Fig. 4. UMAP plots (lower plots) were made using all genes in their respective heat map. These, similar to that in Fig. 4, highlight a graded separation between each of the morphological types. **d.** PCA plot of cells, colored by the 5 morphological PV types, using an extended set of n=124 genes from panels a-c. **e.** Comparison of expression rate of genes in PV-INs from this current versus the CA1-IN study[1], displayed as a heat map. Black line marks loess regression fit. Black points label the 124 svm genes from a-c. Black line marks loess regression fit of all genes, and purple line marks loess regression fit of svm genes. Contrary to the differentially expressed genes in Fig. 4, selected genes followed the expression pattern of all genes (black line), indicating that they did not correspond to unexpected dropouts in the CA1-IN data set. **f.** UMAP based embedding of PV-INs from the CA1-IN study[1], and mapping the PV-INs of this current study onto the UMAP embedding using the 124 svm genes for A-C. Two clusters of mapped cells are shown as determined by K-means clustering. Symbols refer to the following transcriptomic subtypes, as described in the original study: rightward triangle *Pvalb.Tac1.Akr1c18*, leftward triangle *Pvalb.Clql1.Cpne5,* upward triangle *Pvalb.C1ql1.Npy,* downward triangle *Pvalb.Clql1.Pvalb,* plus sign *Pvalb.Tac1.Nr4a2,* open circle *Pvalb.Tac1.Sst,* and star *Pvalb.Tac1.Syt2*. Dashed line separates Pvalb.Tac1 and Pvalb.C1ql types. In conclusion, while for technical reasons the random forest approach revealed a largely different set of genes than pair-wise statistical comparisons between the morphological types, these outcomes corroborate our findings shown in Fig. 4.

Figure S7

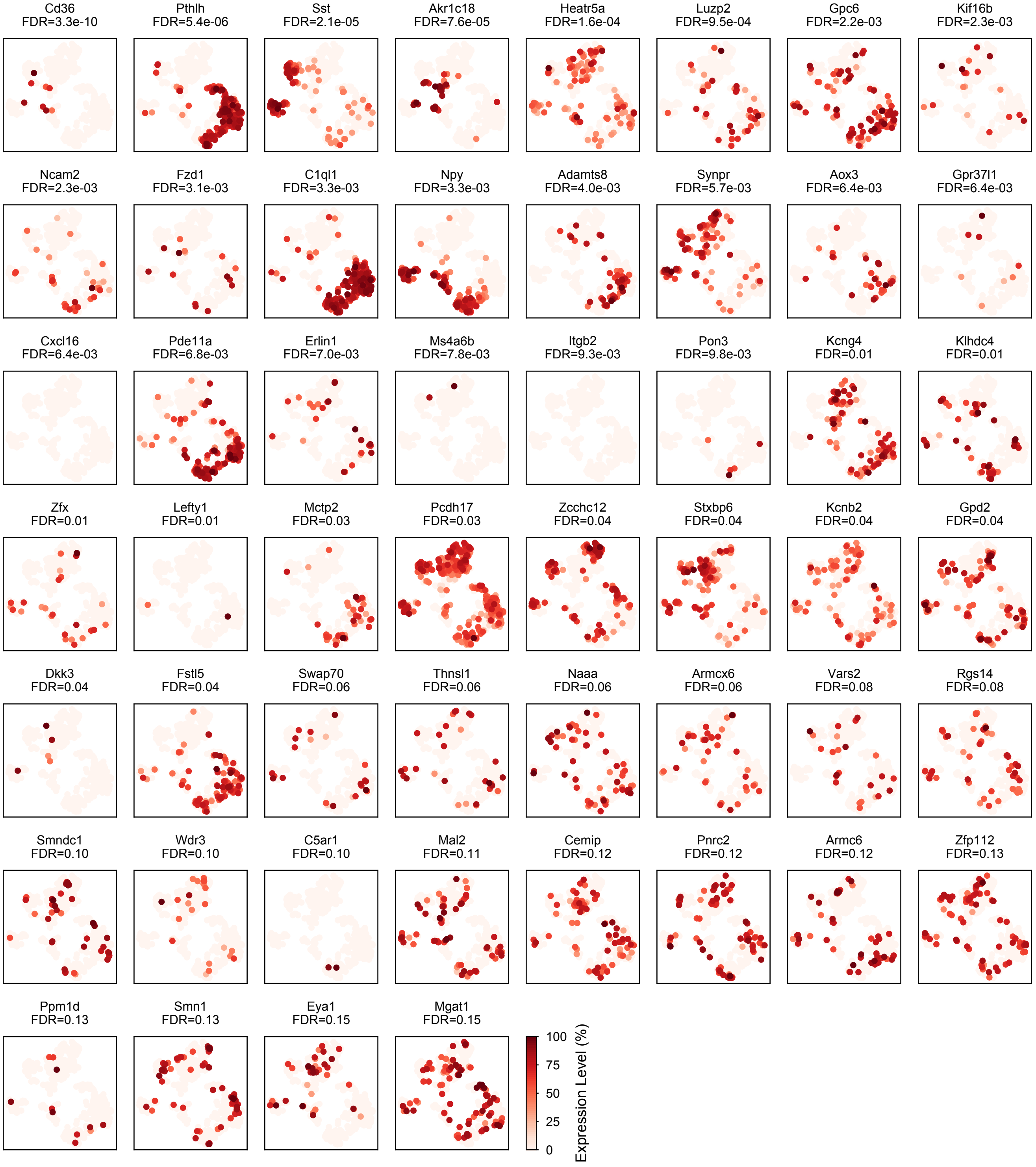
Cd36
FDR=3.3e-10
Pthlh
FDR=5.4e-06
Sst
FDR=2.1e-05
Akr1c18
FDR=7.6e-05
Heatr5a
FDR=1.6e-04
Luzp2
FDR=9.5e-04
Gpc6
FDR=2.2e-03
Kif16b
FDR=2.3e-03
Ncam2
FDR=2.3e-03
Fzd1
FDR=3.1e-03
C1ql1
FDR=3.3e-03
Npy
FDR=3.3e-03
Adamts8
FDR=4.0e-03
Synpr
FDR=5.7e-03
Aox3
FDR=6.4e-03
Gpr37l1
FDR=6.4e-03
Cxcl16
FDR=6.4e-03
Pde11a
FDR=6.8e-03
Erlin1
FDR=7.0e-03
Ms4a6b
FDR=7.8e-03
Itgb2
FDR=9.3e-03
Pon3
FDR=9.8e-03
Kcng4
FDR=0.01
Klhdc4
FDR=0.01
Zfx
FDR=0.01
Lefty1
FDR=0.01
Mctp2
FDR=0.03
Pcdh17
FDR=0.03
Zcchc12
FDR=0.04
Stxbp6
FDR=0.04
Kcnb2
FDR=0.04
Gpd2
FDR=0.04
Dkk3
FDR=0.04
Fstl5
FDR=0.04
Swap70
FDR=0.06
Thnsl1
FDR=0.06
Naaa
FDR=0.06
Armcx6
FDR=0.06
Vars2
FDR=0.08
Rgs14
FDR=0.08
Smndc1
FDR=0.10
Wdr3
FDR=0.10
C5ar1
FDR=0.10
Mal2
FDR=0.11
Cemip
FDR=0.12
Pnrc2
FDR=0.12
Armc6
FDR=0.12
Zfp112
FDR=0.13
Ppm1d
FDR=0.13
Smn1
FDR=0.13
Eya1
FDR=0.15
Mgat1
FDR=0.15
100
75
50
25
0
Expression Level (%)

Fig. S7. Expression of morphology-associated genes in the CA1-IN data set[1]. Using a morphology-based supervised approach, in Fig. 4, we identified a set of n=52 genes, which separated BIC and non-BIC cells, and outlined graded distinctions among the 5 morphological PV types. Using the same UMAP representation as shown in Fig. 4f, we show the expression of each of the morphology-associated 52 genes in the CA1-IN data[1] (each plot is labeled on top with the corresponding gene name). Together, these plots highlight that 2/52 genes were not at all, and further 10/52 genes were detected only in ≤20 of the n=479 PV-INs. As one example, *Akr1c18*, which we found to be differentially expressed between BIC and non-BIC cells, was expressed only in 38/479 cells (7.9%). By contrast, we detected *Akr1c18* in 37/67 cells (55.2%; for completeness, this count also includes BIC type cells, which do not express this gene) in our data set. While the two data set were generated with different approaches, currently there is no cause beyond differences in sequencing depth (10 million versus 0.1 million reads per cell in this and in the CA1-IN study[1]) for this discrepancy.

Figure S8

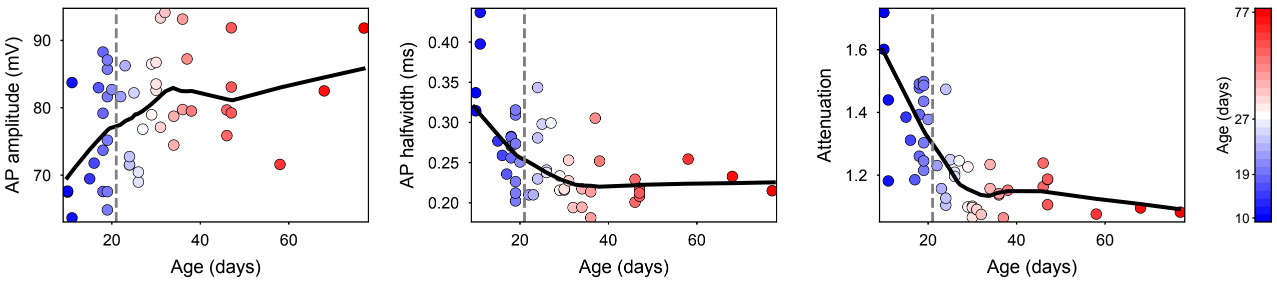

Fig. S8. Electrophysiological analysis of vBC type PV-INs during circuit maturation. In Fig. 6, we identified physiological features in vBC-s, which correlate with age. Plots of three electrophysiological properties (AP amplitude, AP halfwidth, and attenuation, from left to right) that were significantly different (FDR<0.05) between <P21 and >P21 in vBCs versus age. Cells are colored by age and P21 is marked by a dashed gray line. A lowess fit of the distribution is shown in black.

Figure S9

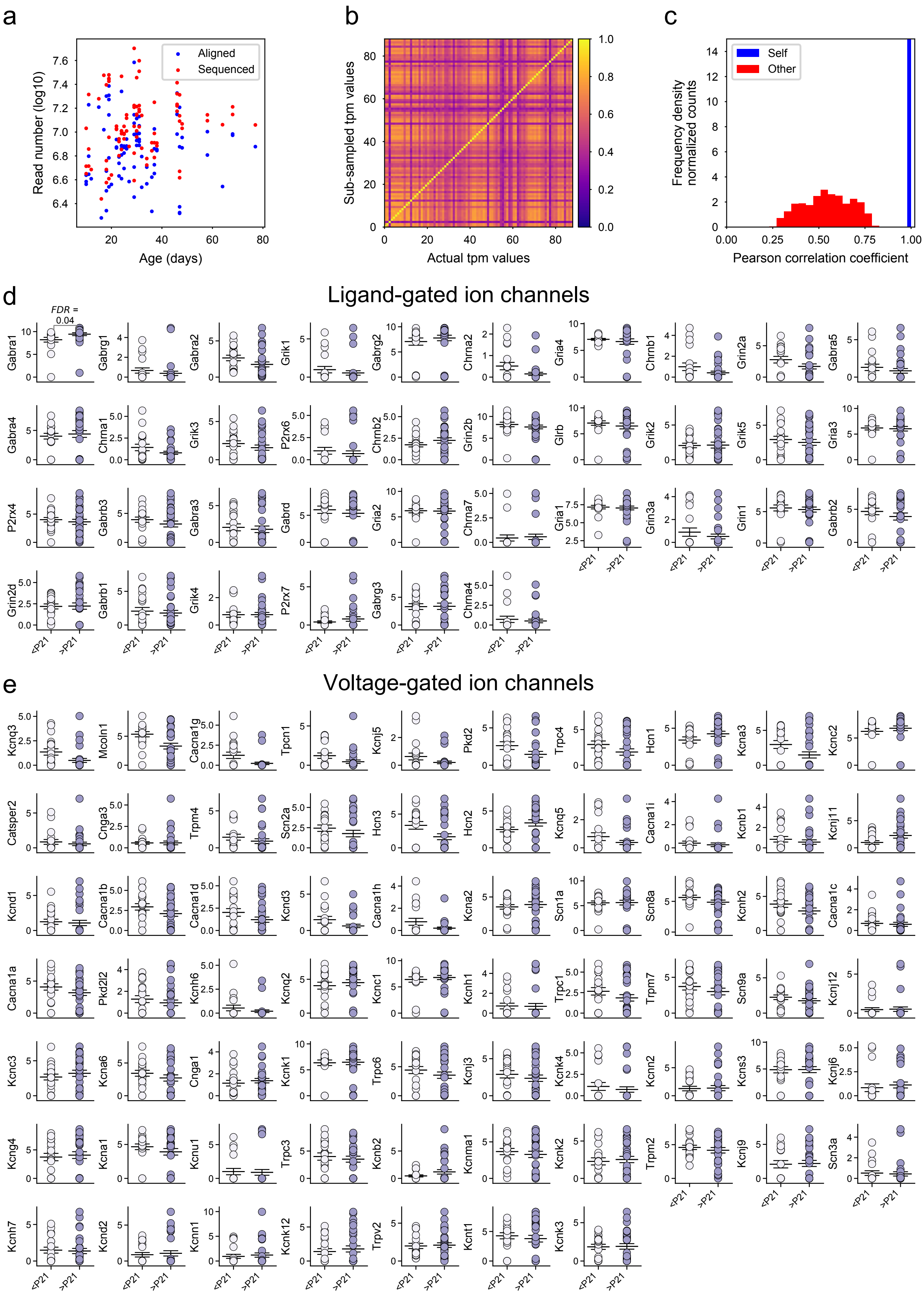

a
Read number (log10)
Age (days)
Aligned
Sequenced
b
Sub-sampled tpm values
Actual tpm values
c
Frequency density normalized counts
Pearson correlation coefficient
Self
Other
d
Ligand-gated ion channels
FDR = 0.04
Gabra1
Gabrg1
Gabra2
Grik1
Gabrg2
Chrna2
Gria4
Chrnb1
Grin2a
Gabra5
Gabra4
Chrna1
Grik3
P2rx6
Chrnb2
Grin2b
Glrb
Grik2
Grik5
Gria3
P2rx4
Gabrb3
Gabra3
Gabrd
Gria2
Chrna7
Gria1
Grin3a
Grin1
Gabrb2
Grin2d
Gabrb1
Grik4
P2rx7
Gabrg3
Chrna4
<P21
>P21
e
Voltage-gated ion channels
Kcnq3
Mcoln1
Cacna1g
Tpcn1
Kcnj5
Pkd2
Trpc4
Hcn1
Kcna3
Kcnc2
Catsper2
Cnga3
Trpm4
Scn2a
Hcn3
Hcn2
Kcnq5
Cacna1i
Kcnb1
Kcnj11
Kcnd1
Cacna1b
Cacna1d
Kcnd3
Cacna1h
Kcna2
Scn1a
Scn8a
Kcnh2
Cacna1c
Cacna1a
Pkd2l2
Kcnh6
Kcnq2
Kcnc1
Kcnh1
Trpc1
Trpm7
Scn9a
Kcnj12
Kcnc3
Kcna6
Cnga1
Kcnk1
Trpc6
Kcnj3
Kcnk4
Kcnn2
Kcns3
Kcnj6
Kcng4
Kcna1
Kcnu1
Trpc3
Kcnb2
Kcnma1
Kcnk2
Trpm2
Kcnj9
Scn3a
Kcnh7
Kcnd2
Kcnn1
Kcnk12
Trpv2
Kcnt1
Kcnk3
<P21
>P21

Fig. S9. Transcriptomic analysis of vBC type PV-INs during circuit maturation. In Fig. 6, we identified physiological features in vBC-s, which correlate with age. Here, we analyzed the expression of ion channel, which define biophysical properties. To begin transcriptomic analyzes (panels a-c), we first run quality controls to compare >P21 data with <P21 data, which latter were largely collected as an extension to the original >P21 data, to confirm that variations, which may occur in sequencing depths in different batches did not introduce transcriptomic noise in our analysis (panels a-c). **a.** Plot shows read depth (number of sequenced, in red, and aligned, in blue, reads) for successive age time-points, revealing that successive time-points do not correlate with read depth. **b.** Before running the TPM normalization, single-cell transcriptomic data were sub-sampled to match the cell with the least number of aligned reads that passed quality control, i.e. we generated a copy of our data in which we randomly sampled the same amount of reads per cell (weighted by the gene distribution in the cell). Plot shows Pearson correlation comparison between the actual TPM values and the sub-sampled TPM values (from 0-1; correlations calculated on Log2(1+TPM), with genes dropped that were expressed in less than 5% of the cells). Cells are ordered according to their age; axis numbers represent cell numbers. This revealed that the sequencing depth was sufficiently large that sub-sampling all cells to the lowest sequencing depth would have negligible effects on the results. **c.** Plot shows the Pearson correlations of cells against their own sub-sampling (blue) and against sub-sampling of other cells (red), revealing a high correlation to themselves and significantly lower ($p=4.7\times10^{-58}$, Mann-Whitney test) correlation to others. Using these post-hoc analyses (panels a-c), it is unlikely that age-dependent variation in sequencing depth has adversely affected our results. **d.** Plots show pair-wise comparison of ligand-gated ion channel gene expression in <P21 (n=19) versus >P21 (n=31) vBC type cells. Each plot is labeled with the name of the corresponding gene. Ordinate values represent normalized $\log_2$(TPM) gene expression level. Abscissa values are labeled only in the bottom, but applicable to all plots. The order of the plots was determined by the FDR value of statistical significance (two-sided Mann-Whitney test), though mean and standard error are shown for each gene. **e.** Plots show pair-wise comparison of voltage-gated ion channel gene expression in <P21 (n=19) versus >P21 (n=31) vBC type cells. The order of the plots was determined by the FDR value of statistical significance (two-sided Mann-Whitney test), though mean and standard error are shown for each gene.

Figure S10

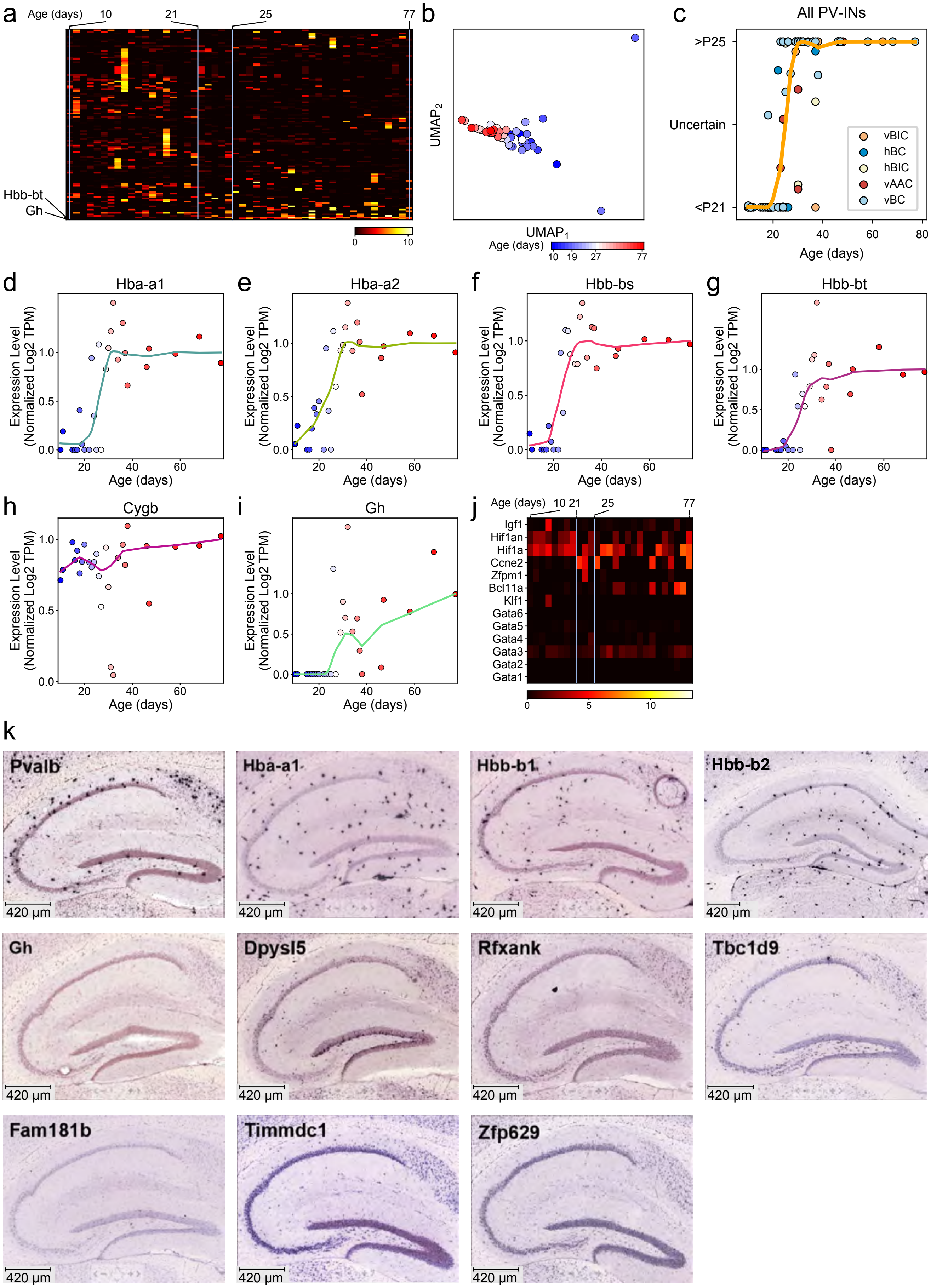
a
Age (days)
10
21
25
77
Hbb-bt
Gh
0
10
b
UMAP2
UMAP1
Age (days)
10 19 27 77
c
All PV-INs
>P25
Uncertain
<P21
vBIC
hBC
hBIC
vAAC
vBC
Age (days)
d
Hba-a1
e
Hba-a2
f
Hbb-bs
g
Hbb-bt
Expression Level
(Normalized Log2 TPM)
Age (days)
h
Cygb
i
Gh
j
Igf1
Hif1an
Hif1a
Ccne2
Zfpm1
Bcl11a
Klf1
Gata6
Gata5
Gata4
Gata3
Gata2
Gata1
k
Pvalb
Hba-a1
Hbb-b1
Hbb-b2
Gh
Dpysl5
Rfxank
Tbc1d9
Fam181b
Timmdc1
Zfp629
420 μm

Fig. S10. Age-dependent gene expression changes in PV-INs. In Fig. 7, we identified rapid transcriptomic changes and an age-dependent onset of hemoglobin mRNA expression in PV-INs. Here, we provide more detailed evidences supporting these findings. **a.** Heat plot shows the expression level of genes identified by Monocle in vBC type cells. GO analysis revealed the presence of multiple gene families, none of which were statistically significant (not shown). Cells (columns) are ordered according to age. **b.** Using the genes identified in panel a, UMAP shows age-dependent separation of vBC type cells. **c.** Plot of consistency with which age can be predicted using Random Forest Classifier based on genes from Fig. 6a, fitted with a loess curve. **d-i.** Normalized loess fits of growth hormone (Gh) and different globin gene expression levels versus cell age in vBC type cells (same loess fits are shown in Fig. 7e). **j.** Heatmap shows the expression of Hb gene regulating factors in vBC type cells, which were sorted by age (left to right). Cells (columns) are ordered according to age. **k.** Photographs show hippocampal ISH data adopted from Allen Mouse Brain Atlas. First, Pvalb (PV) expression is shown for reference. Hb subunits Hba-a1, Hbb-b1, and Hbb-b2 are detected in all samples, which are from P56 male mice (C57BL/6J). Sparsity of expression and localization of cell bodies suggest that Hb expression feasibly occurs in PV-INs. Note that while we found up-regulation of Gh, this ISH data does not provide support for this. Finally, ISH images of down-regulated genes (i.e. *Dpys15*, *Rfxank*, *Tbc1d9*, *Fam181b*, *Timmdc1*, and *Zfp629*) are shown, neither of which suggest expression in the adult mouse CA1.

Figure S11

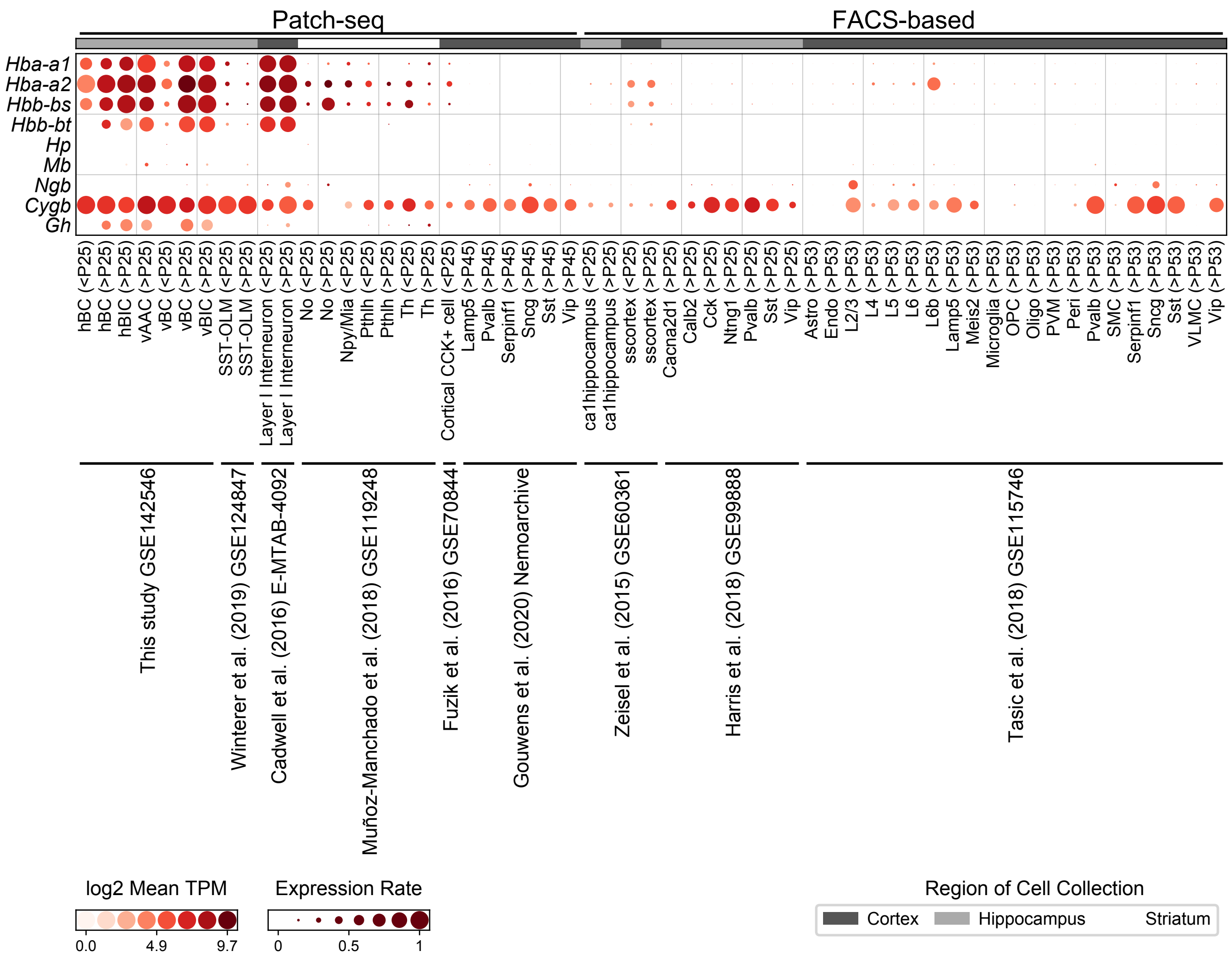

Fig. S11. Detection of hemoglobin mRNA expression in publicly available single-cell RNA-seq datasets. Plot shows average expression level (circle color) and rate (circle size) of hemoglobin related genes and Gh in multiple different data sets. The age of animals (all are mice) and references to the original studies are shown in the bottom, regional origin of cell collection are shown on top. Similar to >P25 hippocampal PV cells, cortical layer 1 interneurons (including neurogliaform and single bouquet cells; from Cadwell et al.[4]) frequently and highly expressed Hb subunits. While Hb subunit expression could be also detected in multiple other cells, their expression rate did not suggest Hb mRNA expression as characteristic feature in other cell types. See Fig. S11 for Hb mRNA expression in single cells at single nucleotide level.

Figure S12

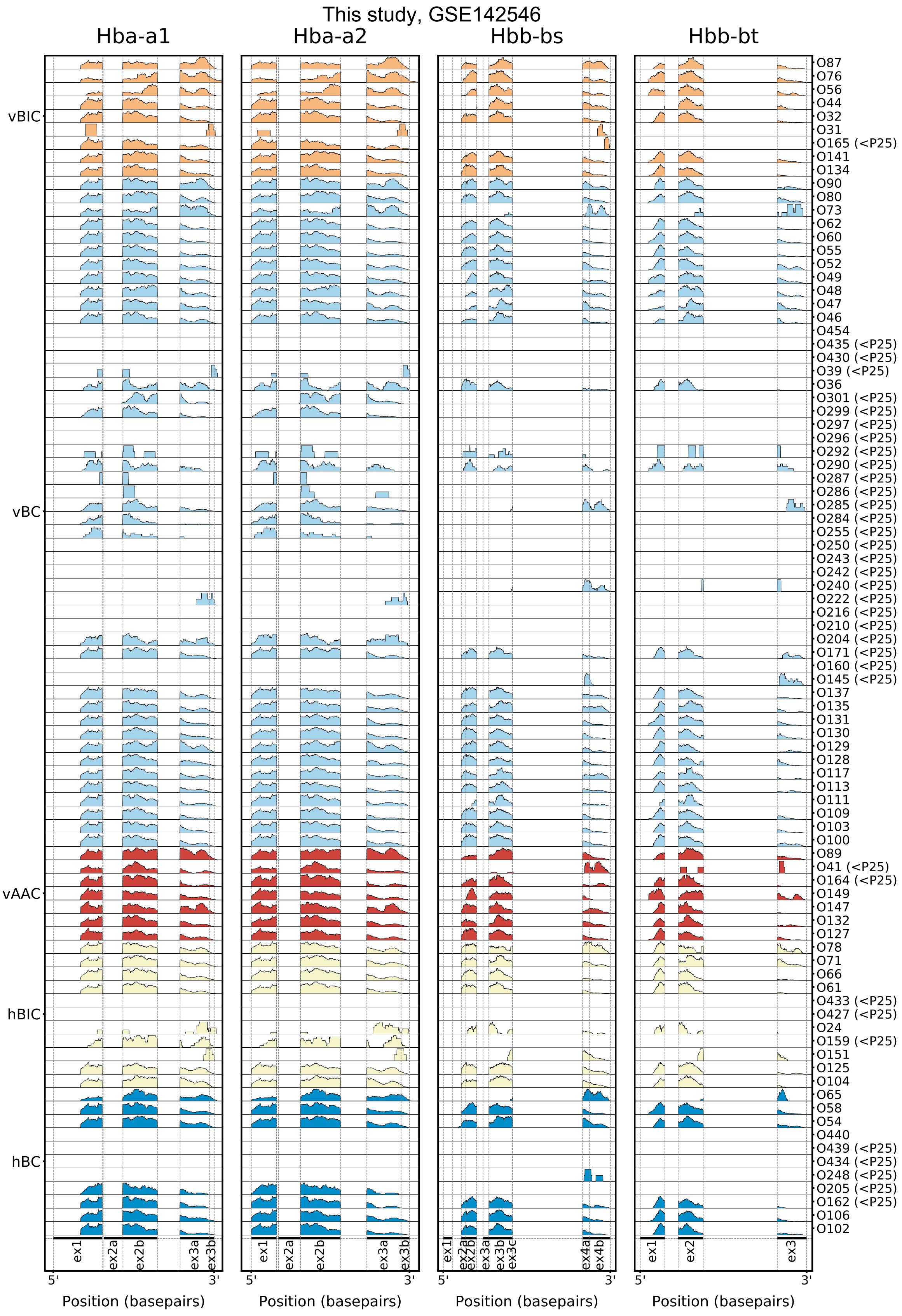
This study, GSE142546
Hba-a1
Hba-a2
Hbb-bs
Hbb-bt
vBIC
vBC
vAAC
hBIC
hBC
O87
O76
O56
O44
O32
O31
O165 (<P25)
O141
O134
O90
O80
O73
O62
O60
O55
O52
O49
O48
O47
O46
O454
O435 (<P25)
O430 (<P25)
O39 (<P25)
O36
O301 (<P25)
O299 (<P25)
O297 (<P25)
O296 (<P25)
O292 (<P25)
O290 (<P25)
O287 (<P25)
O286 (<P25)
O285 (<P25)
O284 (<P25)
O255 (<P25)
O250 (<P25)
O243 (<P25)
O242 (<P25)
O240 (<P25)
O222 (<P25)
O216 (<P25)
O210 (<P25)
O204 (<P25)
O171 (<P25)
O160 (<P25)
O145 (<P25)
O137
O135
O131
O130
O129
O128
O117
O113
O111
O109
O103
O100
O89
O41 (<P25)
O164 (<P25)
O149
O147
O132
O127
O78
O71
O66
O61
O433 (<P25)
O427 (<P25)
O24
O159 (<P25)
O151
O125
O104
O65
O58
O54
O440
O439 (<P25)
O434 (<P25)
O248 (<P25)
O205 (<P25)
O162 (<P25)
O106
O102
ex1
ex2a
ex2b
ex3a
ex3b
ex3c
ex4a
ex4b
ex2
ex3
5'
3'
Position (basepairs)

Figure S12 (Cont.)

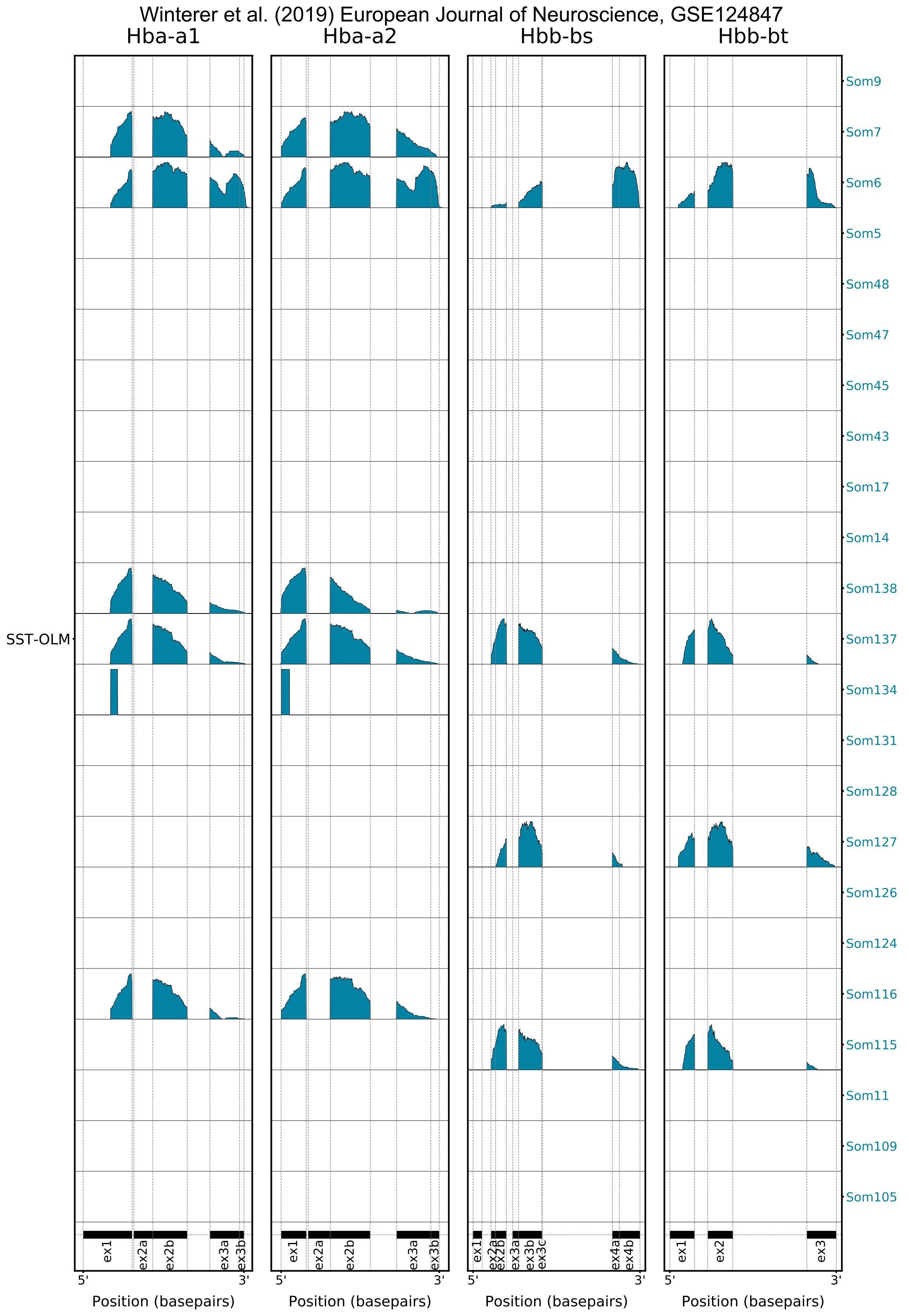

Figure S12 (Cont.)

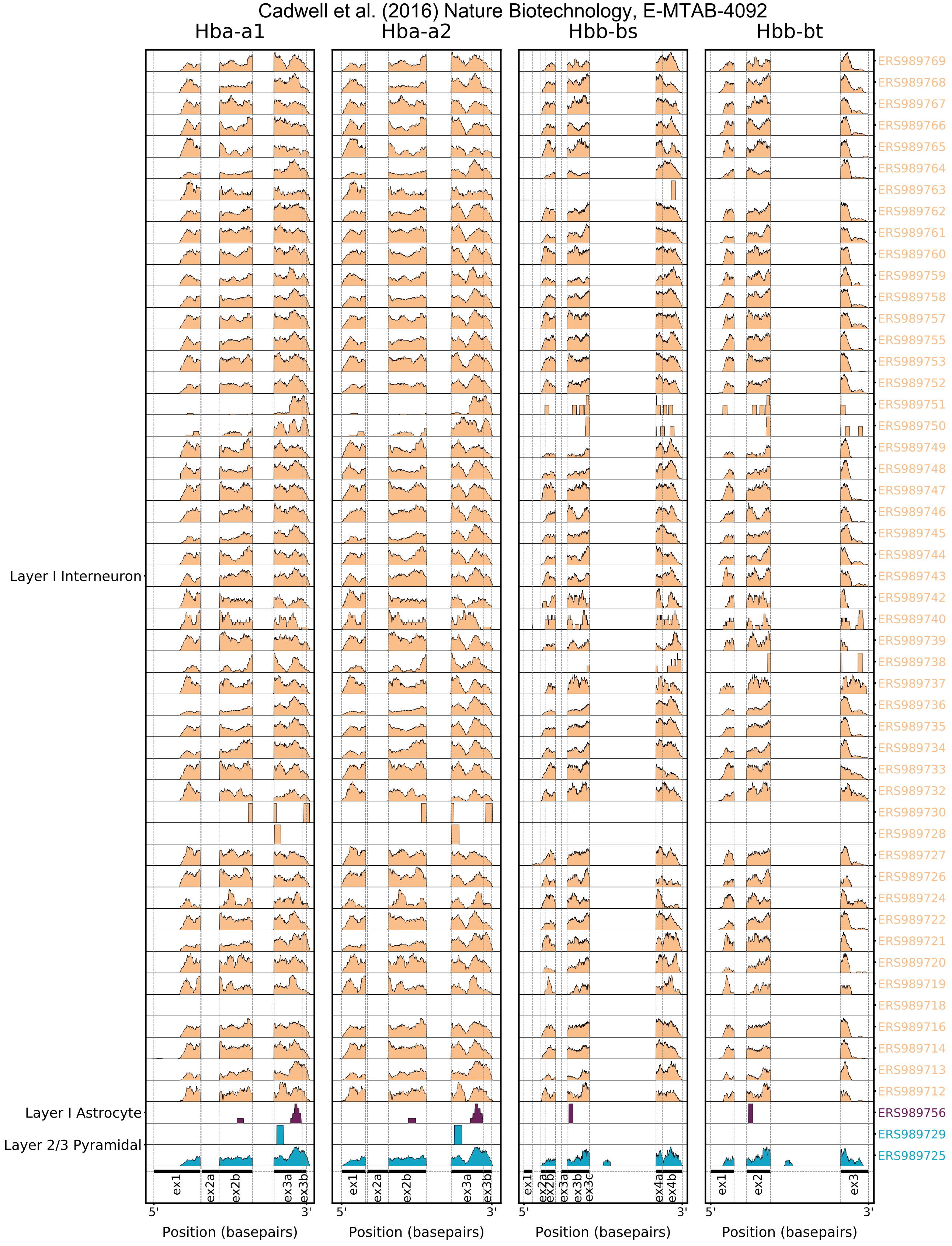

Figure S12 (Cont.)

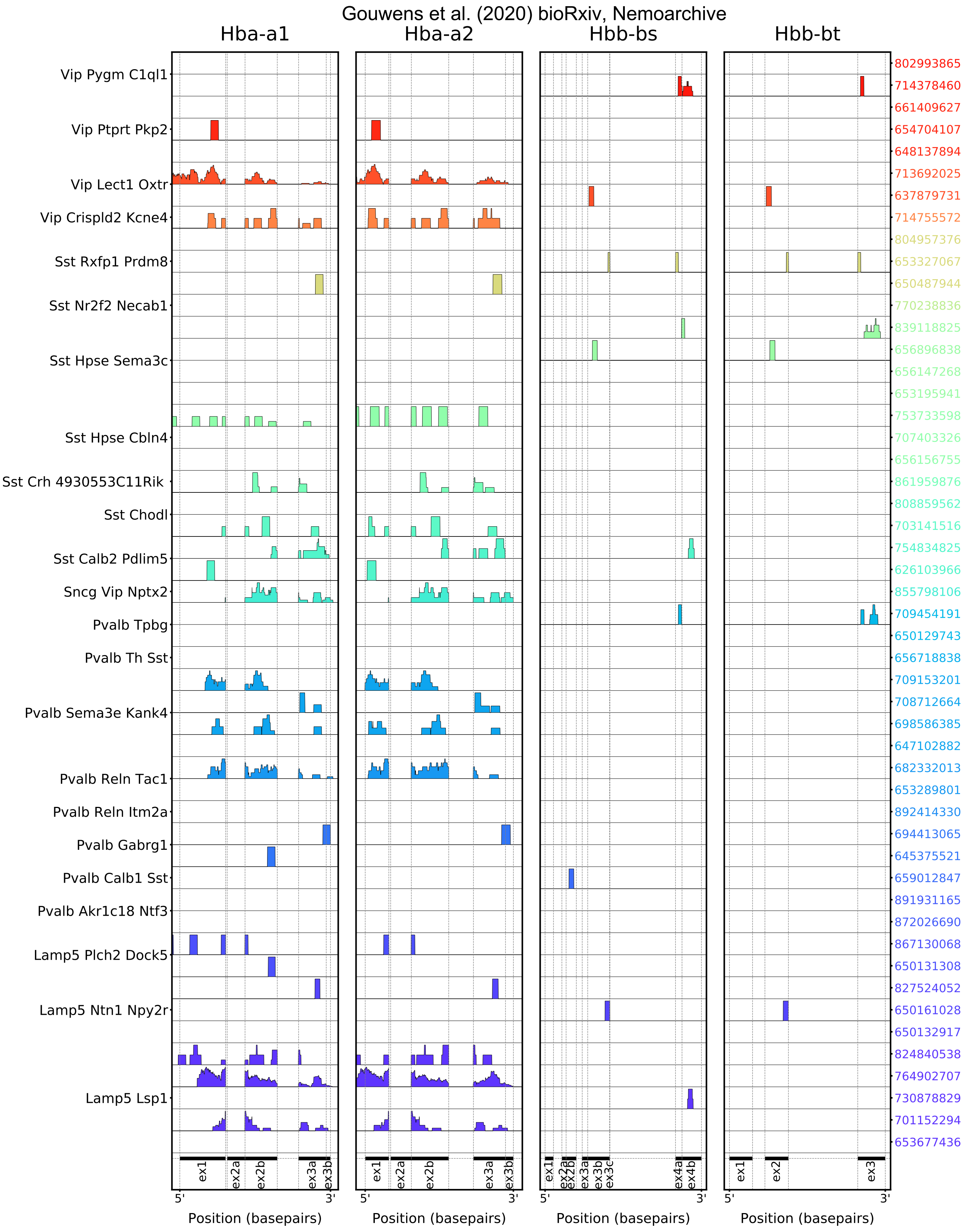

Figure S12 (Cont.)

Tasic et al. (2018) Nature, GSE115746

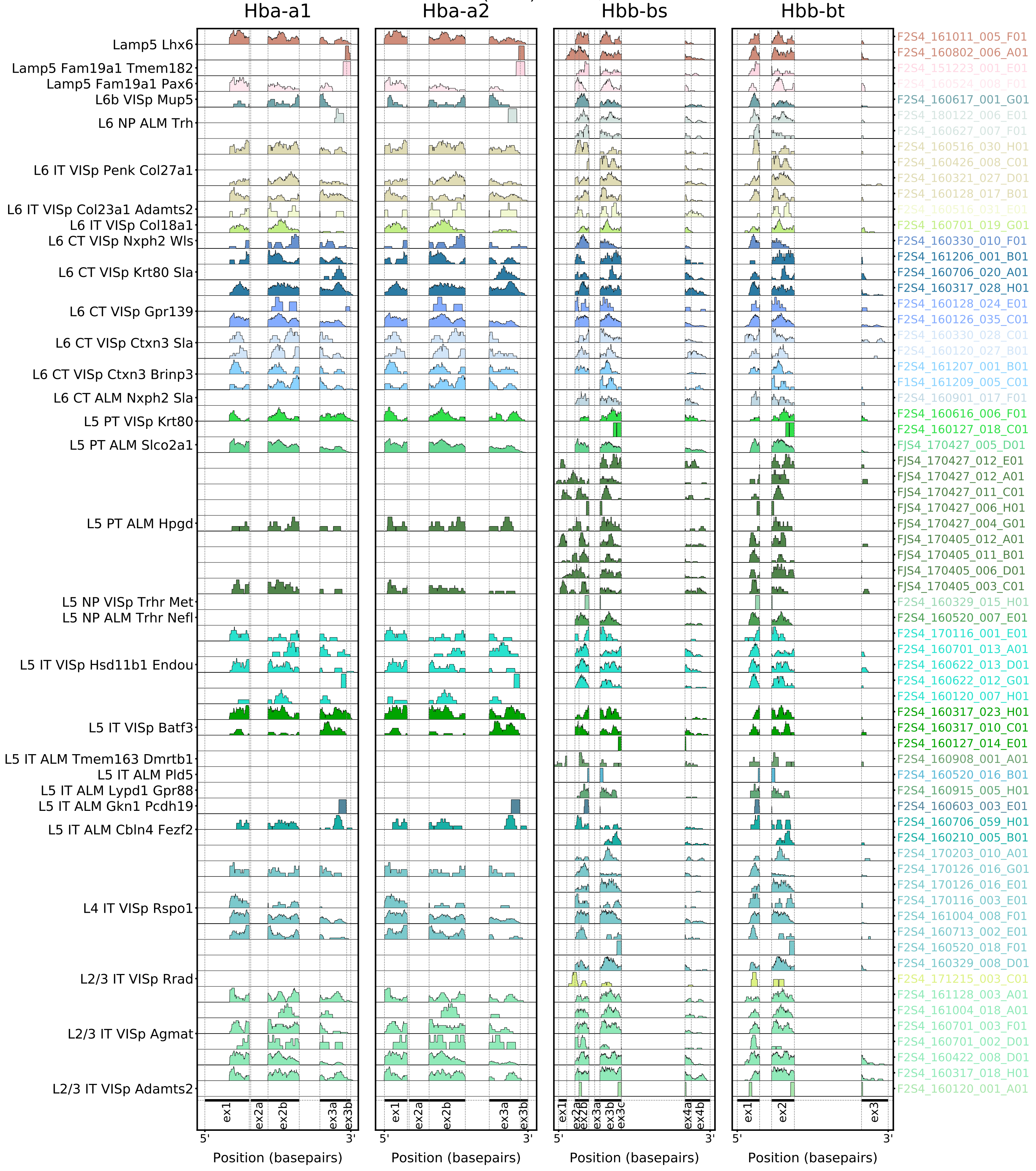

Figure S12 (Cont.)

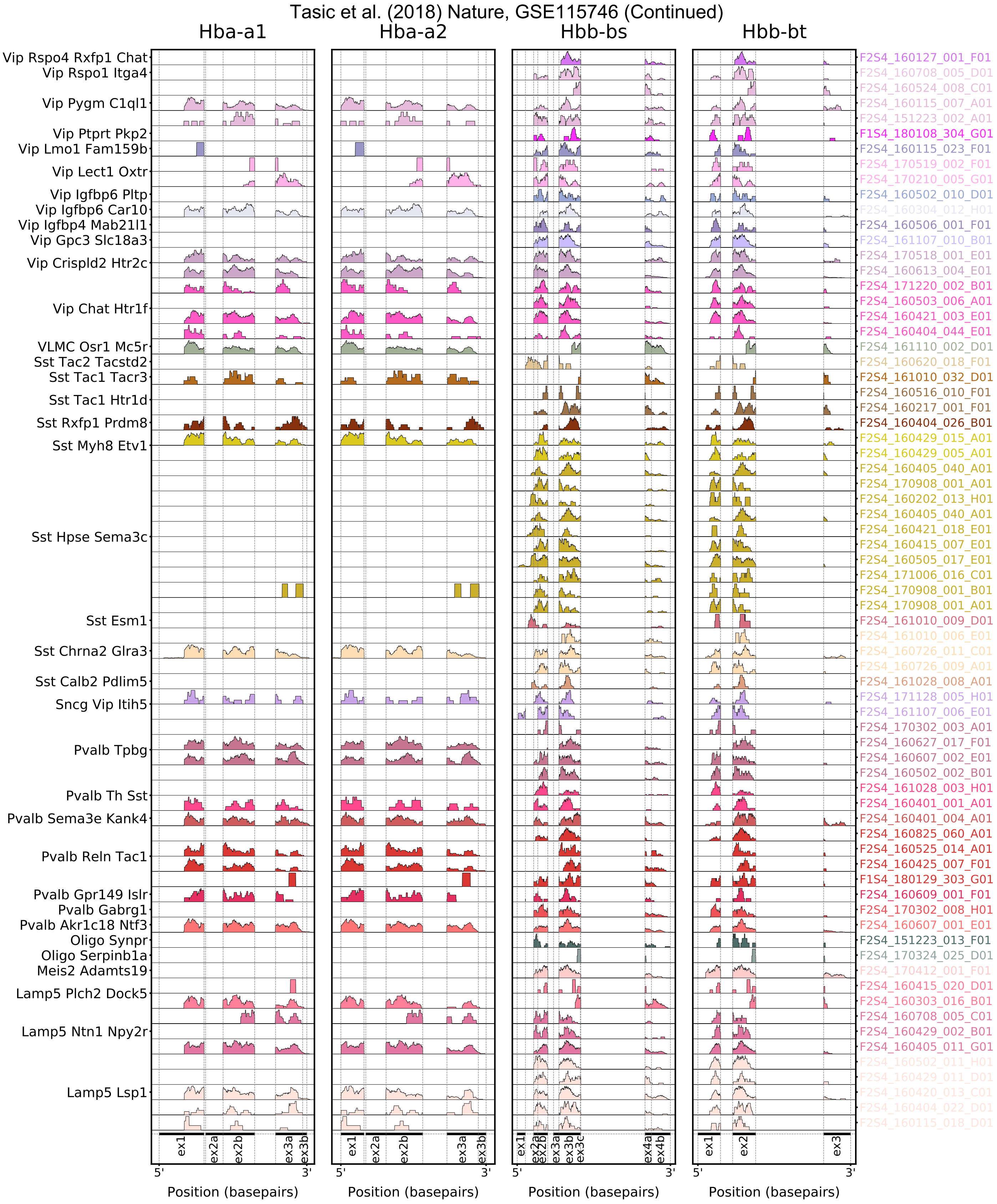

Fig. S12. Hemoglobin subunit expression at single-nucleotide level. Plots show *Hba-a1*, *Hba-a2*, *Hbb-bs* and *Hbb-bt* mRNA expression in single cells at single-nucleotide level (shown data are normalized for each gene and cell). For each data set, reference to the original study (first author, year, journal and data set reference number) is shown on top. Cell types are show on the left, single cell IDs are shown on the right. Exon and intron lengths are shown in the bottom, according to their original scale.

Supplementary References

1. Harris, K.D. et al. Classes and continua of hippocampal CA1 inhibitory neurons revealed by single-cell transcriptomics. *PLoS Biology* **16(6)**, e2006387 (2018).

2. Winterer, J., Lukacsovich, D., Que, L., Sartori, A.M., Luo, W. & Földy, C. Single-cell RNA-Seq characterization of anatomically identified OLM interneurons in different transgenic mouse lines. *Eur. J. Neurosci.* **50(11)**, 3750-3771 (2019).

3. Földy, C., Darmanis, S., Aoto, J., Malenka, R.C., Quake, S.R. & Südhof, T.C. Single-cell RNAseq reveals cell adhesion molecule profiles in electrophysiologically defined neurons. *Proc Natl Acad Sci USA* **113(35)**, E5222-31 (2016).

4. Cadwell, C.R. et al. Electrophysiological, transcriptomic and morphologic profiling of single neurons using Patch-seq. *Nat. Biotechnol.* **34(2)**, 199-203 (2016).
