# Supplementary material for: Transcriptional and morphological profiling of parvalbumin interneuron subpopulations in the mouse hippocampus
Source: Nat Commun. 2021 Jan 4;12:108. doi: 10.1038/s41467-020-20328-4 (PMC7782706; doi:10.1038/s41467-020-20328-4)
Supplement: Supplementary file 3 — Reporting Summary [file 41467_2020_20328_MOESM3_ESM.pdf]

nature research

Corresponding author(s): Csaba Foldy
Last updated by author(s): 09/02/2020

Reporting Summary

Nature Research wishes to improve the reproducibility of the work that we publish. This form provides structure for consistency and transparency in reporting. For further information on Nature Research policies, see our Editorial Policies and the Editorial Policy Checklist.

Statistics

For all statistical analyses, confirm that the following items are present in the figure legend, table legend, main text, or Methods section.

n/a	Confirmed	
☐	☒	The exact sample size (n) for each experimental group/condition, given as a discrete number and unit of measurement
☐	☒	A statement on whether measurements were taken from distinct samples or whether the same sample was measured repeatedly
☐	☒	The statistical test(s) used AND whether they are one- or two-sided<br>*Only common tests should be described solely by name; describe more complex techniques in the Methods section.*
☐	☒	A description of all covariates tested
☐	☒	A description of any assumptions or corrections, such as tests of normality and adjustment for multiple comparisons
☐	☒	A full description of the statistical parameters including central tendency (e.g. means) or other basic estimates (e.g. regression coefficient) AND variation (e.g. standard deviation) or associated estimates of uncertainty (e.g. confidence intervals)
☐	☒	For null hypothesis testing, the test statistic (e.g. F, t, r) with confidence intervals, effect sizes, degrees of freedom and P value noted<br>*Give P values as exact values whenever suitable.*
☒	☐	For Bayesian analysis, information on the choice of priors and Markov chain Monte Carlo settings
☒	☐	For hierarchical and complex designs, identification of the appropriate level for tests and full reporting of outcomes
☐	☒	Estimates of effect sizes (e.g. Cohen's d, Pearson's r), indicating how they were calculated

Our web collection on statistics for biologists contains articles on many of the points above.

Software and code

Policy information about availability of computer code

Data collection	Python 3.7<br>R 3.5.1<br>Kallisto 0.45.1<br>Tximport 1.10.1<br>biomaRt 2.38.0
Data analysis	Python 3.7<br>R 3.5.1<br>edgeR 3.24.3<br>UMAP 0.3.9<br>scikit-learn 0.21.2<br>FIt-SNE 1.1.0<br>nbt-SNE 1.0<br>https://github.com/foldy-lab/Transcriptomics-of-Pvalb-Cells

For manuscripts utilizing custom algorithms or software that are central to the research but not yet described in published literature, software must be made available to editors and reviewers. We strongly encourage code deposition in a community repository (e.g. GitHub). See the Nature Research guidelines for submitting code & software for further information.

Data

Policy information about availability of data

All manuscripts must include a data availability statement. This statement should provide the following information, where applicable:

- Accession codes, unique identifiers, or web links for publicly available datasets
- A list of figures that have associated raw data
- A description of any restrictions on data availability

The datasets generated during and / or analysed during the current study are available in the NCBI GEO repository:
Generated for this study: https://www.ncbi.nlm.nih.gov/geo/query/acc.cgi?acc=GSE142546
Winterer et al. (2019): https://www.ncbi.nlm.nih.gov/geo/query/acc.cgi?acc=GSE124847
Foldy et al. (2016): https://www.ncbi.nlm.nih.gov/geo/query/acc.cgi?acc=GSE75386
Cadwell et al. (2016): https://www.ebi.ac.uk/arrayexpress/experiments/E-MTAB-4092/
Muñoz-Manchado et al. (2018): https://www.ncbi.nlm.nih.gov/geo/query/acc.cgi?acc=GSE119248
Fuzik et al. (2016): https://www.ncbi.nlm.nih.gov/geo/query/acc.cgi?acc=GSE70844
Zeisel et al. (2015): https://www.ncbi.nlm.nih.gov/geo/query/acc.cgi?acc=GSE60361
Harris et al (2018): https://www.ncbi.nlm.nih.gov/geo/query/acc.cgi?acc=GSE99888
Tasic et al. (2018): https://www.ncbi.nlm.nih.gov/geo/query/acc.cgi?acc=GSE115746

Field-specific reporting

Please select the one below that is the best fit for your research. If you are not sure, read the appropriate sections before making your selection.

- [x] Life sciences
- [ ] Behavioural & social sciences
- [ ] Ecological, evolutionary & environmental sciences

For a reference copy of the document with all sections, see nature.com/documents/nr-reporting-summary-flat.pdf

Life sciences study design

All studies must disclose on these points even when the disclosure is negative.

Sample size	Sample size calculation was not used. Post-hoc controls and analyses, e.g. in Fig. S3, were used to determine if differences could be resolved at our sample size.
Data exclusions	Cells that failed a standard quality control test based on number of genes and number of unique genes were excluded from analysis. Both exclusion criteria were determined prior to running the analysis.
Replication	Replication was not applicable to this study. Individual cells belonging to the same morphologically-defined PV subtypes were mostly collected from different mice. To prevent batch effects occurring between different cell types, cells belonging to different morphologically-defined PV subtypes were pooled together in sequencing libraries. In addition, cells were meta-analysed with other publicly available datasets (Harris et al., 2018, Winterer et al., 2019).
Randomization	Individual cells were randomly recorded and collected from randomly chosen mice.
Blinding	Post-hoc staining was used to determine cellular morphology, and therefore the identity of cells was unknown during sample collection.

Reporting for specific materials, systems and methods

We require information from authors about some types of materials, experimental systems and methods used in many studies. Here, indicate whether each material, system or method listed is relevant to your study. If you are not sure if a list item applies to your research, read the appropriate section before selecting a response.

Materials & experimental systems

n/a	Involved in the study	
x		Antibodies
x		Eukaryotic cell lines
x		Palaeontology and archaeology
	x	Animals and other organisms
x		Human research participants
x		Clinical data
x		Dual use research of concern

Methods

n/a	Involved in the study	
x		ChIP-seq
x		Flow cytometry
x		MRI-based neuroimaging

Animals and other organisms

Policy information about studies involving animals; ARRIVE guidelines recommended for reporting animal research

Laboratory animals	Mus Musculus, B6, male and female, ages from P10 to P77 were used in this study. Mice were housed at room temperature, 12-12 hour light cycle, and at 40-60% humidity.
Wild animals	No wild animals were used in the study.
Field-collected samples	No field collected samples were used in the study.
Ethics oversight	All animal protocols and husbandry practices were approved by the Veterinary Office of Zürich Kanton.

Note that full information on the approval of the study protocol must also be provided in the manuscript.
